# Exposure to Oxy-Tetracycline Changes Gut Bacterial Community Composition in Rainbow Trout: A Preliminary Study

**DOI:** 10.3390/ani11123404

**Published:** 2021-11-29

**Authors:** Aritra Roy Choudhury, Ji-Young Park, Do Young Kim, Jeongyun Choi, Satabdi Acharya, Jung-Ho Park

**Affiliations:** 1 Bio-Evaluation Center, Korea Research Institute of Bioscience and Biotechnology, Cheongju 28116, Korea; aritraroy@kribb.re.kr (A.R.C.); pjy125@kribb.re.kr (J.-Y.P.); nemodol2@kribb.re.kr (D.Y.K.); jychoi@kribb.re.kr (J.C.); 2 Department of Bioactive Material Science, College of Natural Science, Jeonbuk National University, Jeonju 54896, Korea; satarya97@gmail.com; 3 Department of Bioprocess Engineering, University of Science and Technology (UST) of Korea, 217 Gajeong-ro, Yuseong-gu, Daejeon 34113, Korea

**Keywords:** antibiotics, oxy-tetracycline, rainbow trout, tenericutes, mollicutes, proteobacteria, metabolic profiles

## Abstract

**Simple Summary:**

The use of antibiotics is extensive in livestock and aquaculture, although various research work is being carried out across the globe for developing alternative technologies. However, the excessive use of antibiotics leads to bio-magnification due to their stability and persistence in the environment. We assessed the gut bacterial diversity of rainbow trout grown at aquacultures in Korea exposed to oxy-tetracycline for the restriction of infections. The study revealed the changes in taxonomic units upon antibiotic treatment and prediction-based functional characterization of the gut bacterial community, providing a general overview of the abundance of particular bacterial taxa.

**Abstract:**

The extensive use of antibiotics is evident in most of the livestock and aquaculture management for inhibiting pathogen infection. Korean aquaculture depends on the usage of oxy-tetracycline for growing rainbow trout. Hence, this study was conducted to evaluate the changes in gut bacterial community profiles of rainbow trout exposed to oxy-tetracycline and predict the metabolic functioning of the bacterial community. The gut bacterial community composition of oxy-tetracycline treated fish was assessed by amplicon sequencing targeting the 16S rRNA gene of bacteria and comparing with the control group that did not receive any antibiotic. The principle coordinate analysis and non-metric multidimensional scaling analysis had shown two distinct clusters that implies the changes in community composition. In phyla level, the relative abundances of Tenericutes and Firmicutes were observed to be significantly higher in oxy-tetracycline treated fish compared to the control. Furthermore, the prediction based metabolic profiling revealed the processes that are affected due to the shift in community profiles. For example, metabolic functioning of membrane efflux system, amino acid metabolism and glycolysis were significantly higher in oxy-tetracycline treated fish compared to the control. This study describes alteration in gut bacterial community composition and potential metabolic profiles of the community that might be responsible for surviving in antibiotic rich environment.

## 1. Introduction

Antibiotic resistance is a growing concern across the globe during the recent past. This is mainly due to the unsupervised usage of broad host range antibiotics maintaining livestock or aquaculture [1]. Aquaculture is common in most of the countries, as fishes are regarded as an important delicacy in almost every culture. Farmers use a wide range of antibiotics to restrict the proliferation of pathogenic bacteria in the water source used for growing fishes [2]. The use of antibiotics for growing fishes has had a significant effect on their intestinal as well as fillet bacterial communities [3,4]. The fish intestine harbors a beneficial microbial community that is known to assist in digestion and disease control [5]. The gut microbiota is also known for enhancing innate immune responses as well as proliferation of epithelial tissue in fishes [6]. Hence, the change in bacterial community composition upon long-term antibiotic exposure might have an adverse effect on the physiological processes of fish.

Rainbow trout are common freshwater fish species that are consumed in various countries, and there are various reports that have revealed the gut bacterial community composition [7,8]. For example, the intestinal microbiome consists majorly of Proteobacteria, Fusobacteria and Firmicutes [7,8]. These bacterial compositions are influenced by various factors such as infection [8], diet [7] and antibiotics [3]. The Korean peninsula is no different in its consumption of a plethora of freshwater as well as marine fishes [9]. The aquacultures in Korea are also using broad host range antibiotics, especially oxy-tetracycline (OTC), on a daily basis to restrict infections [10,11]. However, there is a lack of studies on the characterization of gut microbiota of rainbow trout on long-term exposure to OTC in aquaculture. Hence, this study was conducted to evaluate the shift in gut bacterial community composition of rainbow trout grown in aquaculture and exposed to long-term OTC treatments for controlling pathogen infection. A metagenome-based study was conducted to determine the gut bacterial community composition, and enriched gene encoding enzymes were predicted to correlate the diversity changes and metabolic profiling of the bacterial community.

## 2. Materials and Methods

Rainbow trouts (*Oncorhynchus mykiss*) were cultivated from embryos in the Chugcheongbuk-do Inland Fisheries Research Institute at Republic of Korea. The fish were maintained at 10–13 °C in a 20 L tank and at a density of 20 fish per tank. The fish were fed daily with a formulated feed mixture consisting of 45% crude protein, 6% crude fat, 11.5% ash and 1.5% fibre. OTC was treated (2 mg/20 g fish/day) for 5 days at 90 days after hatching. The gut samples were collected at 24 h after the last OTC treatment. The control and oxytetracycline-treated fish were kept in separate tanks. The dissolved oxygen was maintained at 10–11 mg L^−1^ and pH at 6.7–8.7. About 25% of water from each tank was changed every 2 weeks by using recirculating aquaculture system developed in South Korea. The first segment of the mid-intestine (FSMI) was segregate and immediately stored at −80 °C for DNA extraction. All experiments were performed after receiving approval from the Institutional Animal Care and Use Committee (IACUC) of Korea Research Institute of Bioscience and Biotechnology (approval No: KRIBB-AEC-21275). Metagenomic DNA was extracted using the DNeasy^®^ Blood and Tissue Kit (Qiagen, Valencia, CA, USA). A total of three replicates per treatment were used in this study (treatments: (i) control, (ii) OTC). Each replicate contained a total of three individual fish, and a composite DNA sample per replicate was prepared for sequencing. The extracted DNA was used for high-throughput Illumina MiSeq sequencing at ChunLab, South Korea. The V3-V4 regions of 16S rRNA genes were targeted and amplified using primers 341F (5′-TCGTCGGCAGCTCAGATGTGTATAAGAGACAGCCTACGGGNGGCWGCAG-3′; underlined sequences indicating the target) and 805R (5′-GTCTCGTGGGCTCGGAGATCGTATAAGAGACAGGACTACHVGGTATCTAATCC-3′). The raw data analysis was performed using a Mothur pipeline v. 1.39.5 [12] as per the processes mentioned previously [13]. Briefly, the assembled forward and reverse sequence obtained from the platform were used for discarding the low-quality reads and ambiguous nucleotides for further processing. Chimera were removed using chimera.uchime function, and the taxonomic identity was obtained using Greengenes reference database. The raw sequences were updated in a Sequence Read Archive (SRA) dataset at NCBI (National Center for Biotechnology information) under accession no. SRP341227. Shannon, Simpson and Chao1 indices were calculated in Mothur after the normalization of analyzed data to a minimum number of reads. Principal coordinate analysis (PCoA) and non-metric multidimensional scaling (NMDS) were performed in Mothur. Statistical differences between the means at *p* < 0.05 were determined using Tukey’s test at SAS version 9.4. The predicted metabolic profiles of bacterial communities from the bacterial 16S rRNA abundance data were determined by the PICRUSt2 tool.

## 3. Results

**Figure 1:**
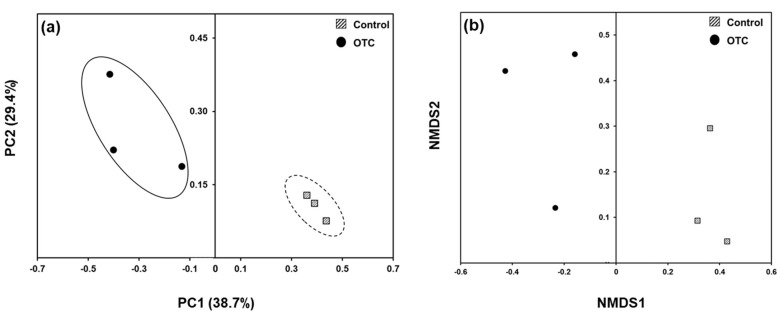
Principal coordinate analysis (PCoA) (**a**) and non-metric multidimensional scaling (NMDS) (**b**) analyses based on Bray–Curtis dissimilatory matrix for gut bacterial community of rainbow trout.

The analysis of sequence data showed goods’ coverage of 99.9% for both the studied rainbow trout groups, which illustrates that the sampling was sufficient for determining the diversity indices. The number of operational taxonomic units (OTUs) did not vary significantly between the control and OTC-treated gut microbiota of rainbow trouts (Table 1). The diversity indices, i.e., shannon and simpson indices, also did not have any significant differences. On the other hand, the richness estimator Chao1 was significantly higher for the gut bacterial composition of control fishes. Two different ordination plot analyses, viz., PCoA and NMDS, were conducted to infer the effect of OTC in gut bacterial community composition of rainbowtrouts (Figure 1). PCoA and NMDS showed two distinct clusterings of gut bacterial community composition. For PCoA, the PC1 axis describes 38.7% variance and PC2 axis describes 29.4% variance. The ordination plots validated the differences in bacterial community composition between two studied groups of fish.

**Figure 2:**
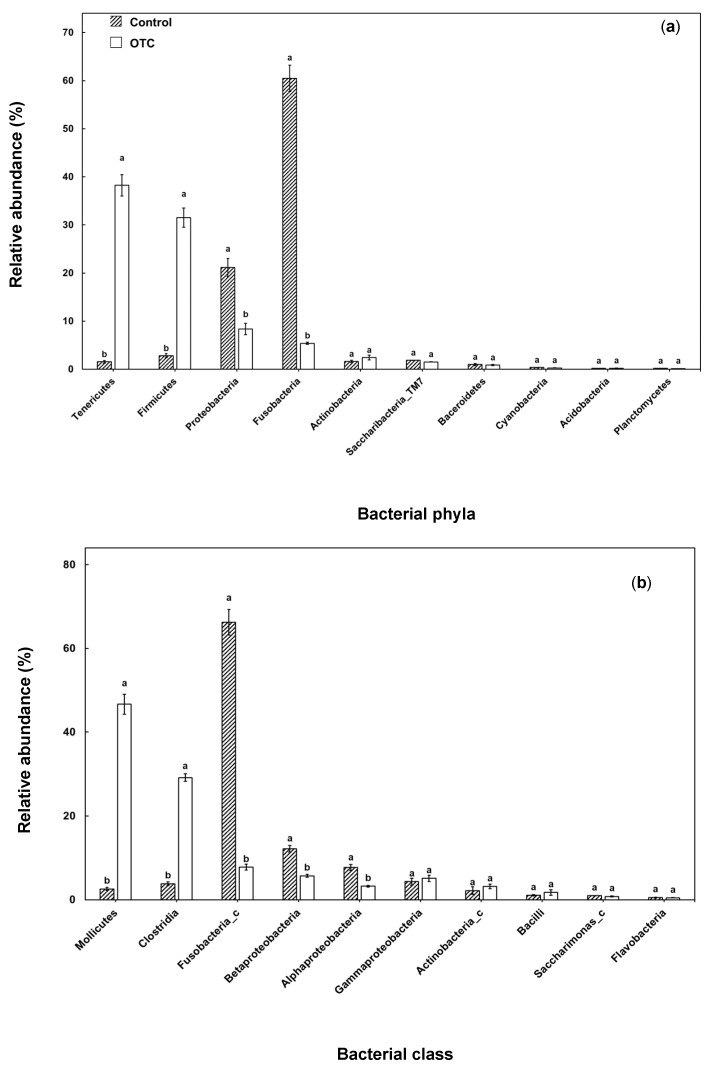
Relative abundances of the 10 most abundant gut bacterial phyla (**a**) and class (**b**) in rainbow trout. Different letters indicate significant differences among taxa at *p* < 0.05 by Tukey’s test.

The taxonomic identities determined from the sequences of the gut of rainbow trouts are depicted at the phylum and class levels for both the studied samples. The relative abundance of 10 most abundant phyla and 20 most abundant bacterial orders are shown in Figure 2. Fusobacteria was observed to be the most abundant phylum in control fish, followed by Proteobacteria, Firmicutes, Tenericutes and Actinobacteria (Figure 2a). On the other hand, the relative abundance of Tenericutes were observed to be the maximum in OTC-treated fish, followed by Firmicutes, Proteobacteria, Fusobacteria and Actinobacteria. The relative abundances of Fusobacteria and Proteobacteria were significantly higher in control fish compared to OTC-treated fish. Conversely, Tenericutes and Firmicutes were significantly higher in OTC-treated fish compared to the control. At class level, the relative abundance of Fusobacteria_c was maximum for control fish, whereas the relative abundance of Mollicutes was maximum for OTC-treated fish (Figure 2b). Furthermore, the relative abundance of Fusobacteria_c, Beta- and Alphaproteobacteria was significantly higher in control fish, and the relative abundance of Mollicutes and Clostridia was maximum for OTC-treated fish compared to the control.

**Figure 3:**
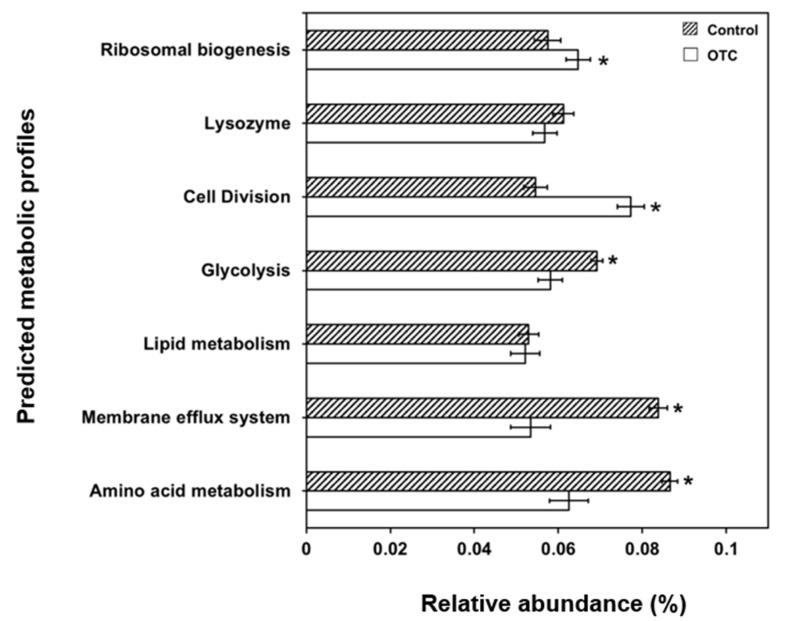
Relative abundance of PICRUSt-derived metabolic profiles of gut bacterial community of rainbow trout. Asterisk (*) denotes *p* < 0.05 by Tukey’s test.

The metabolic profiles were predicted to understand the various metabolic pathways that are affected due to OTC treatment in bacterial community. The PICRUSt predicted metabolic profiles would show a significant increase in functions pertaining to glycolysis, membrane efflux system and amino acid metabolism in OTC-treated fish, compared to the control (Figure 3). On the other hand, the metabolic functions related to cell division and ribosomal biogenesis were observed to be decreased significantly in OTC-treated fish compared to the control fish.

## 4. Discussion

The overall rationale of this study was to determine the gut bacterial diversity of rainbow trout exposed to OTC treatments in Korean aquacultures. A metagenome-based approach was used to evaluate the bacterial diversity and predict the metabolic profiles using the 16S rRNA gene sequencing, and has assisted in understanding the potential changes in gut microbiome of rainbow trout. The long-term use of OTC in aquaculture led to the shift in bacterial community composition as well as changes in metabolic profiles leading to the abundance of specific taxonomic units that can thrive under the influence of broad-spectrum antibiotics.

The OTC treatment did not show any differences among the number of OTUs or diversity indices in the present study, which corroborates a previous report [14], where the OTC treatment on Paralichthys olivaceus did not show any significant differences in the gut bacterial diversity compared to the control group. However, the decrease in richness index can suggest the potential richness of particular groups of bacterial community that are important for survival under antibiotic rich micro-environment [15,16]. These indices depend upon a plethora of factors and cannot provide adequate inferences to community outcomes [17]. Additionally, the diversity indices may not differ from alterations in community composition since differences in abundance of specific taxonomic entities may be compensated for by the differences in abundance of other taxonomic entities [18]. On the other hand, the distinct grouping of both the control as well as the OTC-treated fish reveals the shift in gut bacterial community composition upon OTC treatment. Such a general inference can be observed in other reports, where the fish treated with broad spectrum antibiotics showed distinct clustering of 16S rRNA sequences [19,20]. 

Fusobacteria and Proteobacteria were the two dominant bacterial phyla in control fish that corroborates a previous study [21]. The decrease in Proteobacteria can be attributed to the abundance of fish pathogens that belong to the particular phylum [22,23,24]. Additionally, the decrease in the abundance of Fusobacteria can demonstrate the unregulated effect of OTC in targeting other bacterial groups [25]. However, the abundance of Tenericutes and Firmicutes was higher in OTC-treated fish. Additionally, the related bacterial classes Mollicutes and Clostridia were observed to be in higher abundance in OTC-treated fish. These results corroborate a previous study where the abundance of Tenericutes was higher in *Poecilia reticulata* gut growing in water bodies contaminated with antibiotic discharges from the nearby industries [26]. Tenericutes and its associated class, Mollicutes, are distinct taxonomic units that lack a cell wall that makes easier it for antibiotic resistance genes (ARGs) to be transformed into the bacterial cells [27]. Additionally, the abundance of Firmicutes has been observed in marketable fish that are treated with tetracycline since they possess *tetA* and *tetO* genes [28]. Firmicutes have also been characterized to be abundant in water sediments contaminated with antibiotic residues [29]. Furthermore, a positive correlation has been deduced with the abundance of OTC and Clostridia in environmental samples such as river sediments [29], which might be a proper inference for the Clostridial abundance in OTC-treated fish. In general, OTC treatment has been observed to result in shift in gut bacterial community composition that can be corroborated with the rich antibiotic micro-environment [30].

The variations in the bacterial community composition can affect the metabolic profiles of the bacterial community, as observed from the PICRUSt derived predictions. The metabolic profiles related to ribosomal biogenesis and cell division were significantly lower in OTC-treated fish compared to the control. Such observations can be attributed to decrease in cellular processes of gut microbiome of fish upon exposure to OTC [31], leading to decrease in ribosomal biogenesis and cell division. On the other hand, the increase in membrane efflux system and amino acid metabolism are atypical for gut bacterial diversity of fish upon antibiotic treatments [26,32]. The increase in abundance of membrane efflux systems can assist in expelling OTC from the intra-cellular milieu of bacteria and correlate to their survival in antibiotic rich environmental niche [26]. Additionally, the translation of higher amino acid residues is important for increasing the expression of membrane efflux systems upon antibiotic treatments in fish [33]. The increase in glycolytic pathway in this study contradicts the previous report where OTC treatment in zebrafish predicted a decrease in glycolysis/gluconeogenesis and oxidative phosphorylation of the gut bacterial community [33]. However, it can be proposed that the mechanism of action of gut microbial community might be distinct for different species, and in the present scenario the glycolytic pathway can be assisting in providing ATP for amino acid metabolism as well as functioning of membrane efflux system. The overall study gives a general idea regarding the changes in gut bacterial community composition due to OTC treatment in rainbow trout, and further studies concentrating on designing alternative approaches [34] will be helpful in reducing the use of antibiotics in aquaculture.

## 5. Conclusions

The gut bacterial community profiles of rainbow trout altered upon exposure to tetracycline. The relative abundances of few bacterial taxa, namely, Tenericutes and Firmicutes, were significantly higher in OTC-treated fish compared to the control. These bacterial taxa have the tendency to accumulate antibiotic resistance genes and survive in an antibiotic rich environment due to their distinct characteristics. Moreover, the predicted metabolic profiles revealed the various functions of gut bacterial community to thrive in an antibiotic rich environment. Hence, the collective data represent the potential shift in gut microbial taxonomic units of rainbow trout exposed to broad spectrum antibiotics in aquacultures. Further studies about the correlation of such microbial community changes in physiological and biochemical responses in fish would give a broader account of the effect of antibiotics in aquaculture.

## Figures and Tables

**Table 1:** Diversity indices of studied rainbow trout gut samples.

Treatments	Goods’ Coverage (%)	No. of OTUs	Shannon	Chao1	Simpson
Control	99.9	336 ± 47.88 ^a^	1.72 ± 0.54 ^a^	277 ± 32.77 ^a^	0.42 ± 0.18 ^a^
OTC	99.9	278 ± 24.09 ^a^	1.13 ± 0.59 ^a^	211 ± 20.44 ^b^	0.63 ± 0.19 ^a^

Each value represents the mean ± standard error (number of replicates = 3). Different letters indicate significant differences between the treatments at *p* < 0.05 according to Tukey’s test.

## Data Availability

The data and analyses from the current study are available from the corresponding author upon reasonable request. The raw reads were deposited in SRA archives and can be accessed by accession no. SRP341227.

## References

[1] Zhao Y., Yang Q.E., Zhou X., Wang F.H., Muurinen J., Virta M.P., Brandt K.K., Zhu Y.G. (2021). Antibiotic resistome in the livestock and aquaculture industries: Status and solutions. Crit. Rev. Environ. Sci. Technol..

[2] Miranda C.D., Godoy F.A., Lee M.R. (2018). Current status of the use of antibiotics and the antimicrobial resistance in the Chilean salmon farms. Front. Microbiol..

[3] Merrifield D.L., Bradley G., Baker R.T.M., Davies S.J. (2010). Probiotic applications for rainbow trout (*Oncorhynchus mykiss* Walbaum) II. Effects on growth performance, feed utilization, intestinal microbiota and related health criteria postantibiotic treatment. Aquac. Nutr..

[4] Shehata H.R., Mitterboeck T.F., Hanner R. (2020). Characterization of the microbiota of commercially traded finfish fillets. Food Res. Int..

[5] de Bruijn I., Liu Y., Wiegertjes G.F., Raaijmakers J.M. (2018). Exploring fish microbial communities to mitigate emerging diseases in aquaculture. FEMS Microbiol. Ecol..

[6] Gómez G.D., Balcázar J.L. (2008). A review on the interactions between gut microbiota and innate immunity of fish. FEMS Microbiol. Ecol..

[7] Wong S., Waldrop T., Summerfelt S., Davidson J., Barrows F., Kenney P.B., Welch T., Wiens G.D., Snekvik K., Rawls J.F. (2013). Aquacultured rainbow trout (*Oncorhynchus mykiss*) possess a large core intestinal microbiota that is resistant to variation in diet and rearing density. Appl. Environ. Microbiol..

[8] Parshukov A.N., Kashinskaya E.N., Simonov E.P., Hlunov O.V., Izvekova G.I., Andree K.B., Solovyev M.M. (2019). Variations of the intestinal gut microbiota of farmed rainbow trout, *Oncorhynchus mykiss* (Walbaum), depending on the infection status of the fish. J. Appl. Microbiol..

[9] Yim U.H., Hong S.H., Shim W.J., Oh J.R. (2005). Levels of persistent organochlorine contaminants in fish from Korea and their potential health risk. Arch. Environ. Contam. Toxicol..

[10] Kim W., Lee Y., Kim S.D. (2017). Developing and applying a site-specific multimedia fate model to address ecological risk of oxytetracycline discharged with aquaculture effluent in coastal waters off Jangheung, Korea. Ecotoxicol. Environ. Saf..

[11] Kang H.S., Lee S.B., Shin D., Jeong J., Hong J.H., Rhee G.S. (2018). Occurrence of veterinary drug residues in farmed fishery products in South Korea. Food Control.

[12] Schloss P.D., Westcott S.L., Ryabin T., Hall J.R., Hartmann M., Hollister E.B., Lesniewski R.A., Oakley B.B., Parks D.H., Robinson C.J. (2009). Introducing mother: Opensource, platform-independent, community supported software for describing and comparing microbial communities. Appl. Environ. Microbiol..

[13] Samaddar S., Chatterjee P., Truu J., Anandam R., Kim S., Sa T. (2019). Long-term phosphorus limitation changes the bacterial community structure and functioning in paddy soils. Appl. Soil Ecol..

[14] Kim A., Kim N., Roh H.J., Chun W.K., Ho D.T., Lee Y., Kim D.H. (2019). Administration of antibiotics can cause dysbiosis in fish gut. Aquaculture.

[15] Rasul M.G., Majumdar B.C. (2017). Abuse of antibiotics in aquaculture and it’s effects on human, aquatic animal and environment. Soudi J. Life Sci..

[16] Yukgehnaish K., Kumar P., Sivachandran P., Marimuthu K., Arshad A., Paray B.A., Arockiaraj J. (2020). Gut microbiota metagenomics in aquaculture: Factors influencing gut microbiome and its physiological role in fish. Rev. Aquac..

[17] Shade A. (2017). Diversity is the question, not the answer. ISME J..

[18] Hartmann M., Widmer F. (2006). Community structure analyses are more sensitive to differences in soil bacterial communities than anonymous diversity indices. Appl. Environ. Microbiol..

[19] Almeida A.R., Tacão M., Machado A.L., Golovko O., Zlabek V., Domingues I., Henriques I. (2019). Long-term effects of oxytetracycline exposure in zebrafish: A multi-level perspective. Chemosphere.

[20] Rosado D., Xavier R., Severino R., Tavares F., Cable J., Pérez-Losada M. (2019). Effects of disease, antibiotic treatment and recovery trajectory on the microbiome of farmed seabass (*Dicentrarchus labrax*). Sci. Rep..

[21] Michl S.C., Ratten J.M., Beyer M., Hasler M., LaRoche J., Schulz C. (2017). The malleable gut microbiome of juvenile rainbow trout (*Oncorhynchus mykiss*): Diet-dependent shifts of bacterial community structures. PLoS ONE.

[22] Smith P., Hiney M.P., Samuelsen O.B. (1994). Bacterial resistance to antimicrobial agents used in fish farming: A critical evaluation of method and meaning. Annu. Rev. Fish Dis..

[23] Kholil M.I., Hossain M.M.M., Neowajh M.S., Islam M.S., Kabir M. (2015). Comparative efficiency of some commercial antibiotics against Pseudomonas infection in fish. Int. J. Fish. Aquat..

[24] Serrano P.H. (2005). Responsible use of antibiotics in aquaculture. FAO Fisheries Technical Paper.

[25] Payne C.J., Turnbull J.F., MacKenzie S., Crumlish M. (2021). Investigating the Effect of an Oxytetracycline Treatment on the Gut Microbiome and Antimicrobial Resistance Gene Dynamics in Nile Tilapia (*Oreochromis niloticus*). Antibiotics.

[26] Jia J., Gomes-Silva G., Plath M., Pereira B.B., UeiraVieira C., Wang Z. (2021). Shifts in bacterial communities and antibiotic resistance genes in surface water and gut microbiota of guppies (*Poecilia reticulata*) in the upper Rio Uberabinha, Brazil. Ecotoxicol. Environ. Saf..

[27] Ding C., Yang D., Ma J., Jin M., Shen Z., Shi D., Tian Z., Kang M., Li J., Qiu Z. (2020). Effects of free antibiotic resistance genes in the environment on intestinal microecology of mice. Ecotoxicol. Environ. Saf..

[28] Yuan L., Wang L., Li Z.H., Zhang M.Q., Shao W., Sheng G.P. (2019). Antibiotic resistance and microbiota in the gut of Chinese four major freshwater carp from retail markets. Environ. Pollut..

[29] Guan Y., Jia J., Wu L., Xue X., Zhang G., Wang Z. (2018). Analysis of bacterial community characteristics, abundance of antibiotics and antibiotic resistance genes along a pollution gradient of Ba River in Xi’an, China. Front. Microbiol..

[30] Navarrete P., Mardones P., Opazo R., Espejo R., Romero J. (2008). Oxytetracycline treatment reduces bacterial diversity of intestinal microbiota of Atlantic salmon. J. Aquat. Anim. Health.

[31] Qian M., Wang J., Ji X., Yang H., Tang B., Zhang H., Yang G., Bao Z., Jin Y. (2021). Sub-chronic exposure to antibiotics doxycycline, oxytetracycline or florfenicol impacts gut barrier and induces gut microbiota dysbiosis in adult zebrafish (*Daino rerio*). Ecotoxicol. Environ. Saf..

[32] Kokou F., Sasson G., Mizrahi I., Cnaani A. (2020). Antibiotic effect and microbiome persistence vary along the European seabass gut. Sci. Rep..

[33] Almeida A.R., Alves M., Domingues I., Henriques I. (2019). The impact of antibiotic exposure in water and zebrafish gut microbiomes: A 16S rRNA gene-based metagenomic analysis. Ecotoxicol. Environ. Saf..

[34] Vargas-Albores F., Martínez-Córdova L.R., Hernández-Mendoza A., Cicala F., Lago-Lestón A., Martínez-Porchas M. (2021). Therapeutic modulation of fish gut microbiota, a feasible strategy for aquaculture?. Aquaculture.

